# Prestigious Science Journals Struggle to Reach Even Average Reliability

**DOI:** 10.3389/fnhum.2018.00037

**Published:** 2018-02-20

**Authors:** Björn Brembs

**Affiliations:** Institute of Zoology—Neurogenetics, Universität Regensburg, Regensburg, Germany

**Keywords:** journals, journal ranking, reliability, reproducibility of results, science policy

## Abstract

In which journal a scientist publishes is considered one of the most crucial factors determining their career. The underlying common assumption is that only the best scientists manage to publish in a highly selective tier of the most prestigious journals. However, data from several lines of evidence suggest that the methodological quality of scientific experiments does not increase with increasing rank of the journal. On the contrary, an accumulating body of evidence suggests the inverse: methodological quality and, consequently, reliability of published research works in several fields may be *decreasing* with increasing journal rank. The data supporting these conclusions circumvent confounding factors such as increased readership and scrutiny for these journals, focusing instead on quantifiable indicators of methodological soundness in the published literature, relying on, in part, semi-automated data extraction from often thousands of publications at a time. With the accumulating evidence over the last decade grew the realization that the very existence of scholarly journals, due to their inherent hierarchy, constitutes one of the major threats to publicly funded science: hiring, promoting and funding scientists who publish unreliable science eventually erodes public trust in science.

## Introduction

The most groundbreaking, transformative research results deserve a broad readership and a large audience. Therefore, scientists submit their best work to the journals with the largest audience. While the number of scientists has been growing exponentially over the last decades, the number of journals with a large audience has not kept up, neither has the number of articles published per journal. Consequently, rejection rates at the most prestigious journals has fallen below 10% and the labor of rejecting submissions has become these journals’ largest cost item. Assuming that this exclusivity allows the journals to separate the wheat from the chaff, successful publication in these journals is treated as a quality signal in hiring, promotion and funding decisions. If anything, these developments have fueled the circularity of this relationship: today, publishing ground-breaking science in a high ranking journals is not only important for science to advance but also for an author’s career to advance. Even before science became hypercompetitive at every level, now and again results published in prestigious journals were later found to be false. This is the nature of science. Science is difficult, complicated and perpetually preliminary. Science is self-correcting and better experimentation will continue to advance science to the detriment of previous experiments. Today, however, fierce competition exacerbates this trait and renders it a massive problem for scholarly journals. Now it has become their task to find the ground-breaking among the too-good-to-be-true data, submitted by desperate scientists, who face unemployment and/or laboratory closure without the next high-profile publication. This is a monumental task, given that sometimes it takes decades to find that one or the other result rests on flimsy grounds. How is our hierarchy of more than 30,000 journals holding up?

At first glance, it appears as if our journals fail miserably. Evaluating retractions, the capital punishment for articles found to be irreproducible, it was found that the most prestigious journals boast the largest number (Fang and Casadevall, 2011) and that most of these retractions are due to fraud (Fang et al., 2012). However, data on retractions suffer from two major flaws which make them rather useless for answering questions about the contribution of journals to the reliability (or lack thereof) of our scholarly literature: (1) retractions cover only about 0.05% of the literature; and (2) they are confounded by error-detection variables that are hard to trace. So may be our journals are not doing so horribly after all?

## Journal Ranking

The most widely used metric to rank journals is Clarivate Analytics’ “Impact Factor” (IF), a measure based loosely on citations. Despite the numerous flaws described (e.g., Moed and van Leeuwen, 1996; Seglen, 1997; Saha et al., 2003; Rossner et al., 2007; Adler et al., 2009; Hernán, 2009; Vanclay, 2011; Brembs et al., 2013), the IF is an excellent and consistent descriptor of subjective journal hierarchy, i.e., the level of prestige scientists ascribe to the journals in their respective fields (Gordon, 1982; Saha et al., 2003; Yue et al., 2007; Sønderstrup-Andersen and Sønderstrup-Andersen, 2008). That a measure so flawed still conforms to the expectations of the customers expected to pay for it, is remarkable in its own right. Due to this consistency, the IF is used here as a measure for the subjective ranking of journals by the scientists using these journals: to what extent is this subjective notion of prestige warranted, based on the available evidence? Are prestigious journals really better at detecting the real breakthrough science in the sea of seemingly breakthrough science than average journals?

## Retractions and Error Detection

If anything, one could tentatively interpret what scant data there are on retractions, as suggestive that increased scrutiny may only play a minor role in a combination of several factors leading to more retractions in higher ranking journals.

For instance, there are low ranking journals with high retraction rates (Fang et al., 2012), showing that the involved parties are motivated to retract articles even in low ranking journals. In fact, in absolute terms, most retracted articles come from low-ranking journals. This would be difficult to explain if low ranking journals were less willing to retract and/or scholars less motivated to pursue retractions from these journals. On the other hand, one can make the claim that the numbers would show even more retractions in low ranking journals, if the motivation and willingness to retract were equal. As neither willingness of journals to retract nor motivation in individuals to force a retraction can be quantified, all that the data can show is that it is not an all-or-nothing effect: there is both willingness and motivation to retract also for articles in lower ranking journals.

Another reason why scrutiny might be assumed to be higher in more prestigious journals is that readership is higher, leading to more potential for error detection. More eyes are more likely to detect potential errors. The consequence of this reasonable and plausible factor is difficult to test empirically. However, one could make a more easily testable, analogous claim, such as that one would also expect increased readership to lead to a higher potential not only for retractions but also for citations. More eyes are more likely to detect a finding worth citing. In fact, if anything, citations ought to correlate better with journal rank than retractions because citing an article in a leading journal is not only technically easier than forcing a retraction, it also benefits one’s own research by elevating the perceived importance of one’s own field. However, the opposite is the case: The coefficient of determination for citations with journal rank currently lies around 0.2, while that coefficient comes to lie at just under 0.8 for retractions and journal rank (Brembs et al., 2013). So while there may be a small effect of scrutiny/motivation, the evidence seems to suggest that it is a relatively minor effect, if there is one at all.

Taken together, there is currently no strong case to be made as to whether the likely increased scrutiny and readership of highly-ranked journals is a major factor driving retractions or not. If that were the case, it would indicate that the apparent increased unreliability in high-ranking journals is merely an artifact of the increased scrutiny to retract, combined with an increased willingness of these journals to correct the scientific record. At least two lines of inquiry did not turn up any conclusive evidence for such an argument. With such unclarified confounds in such a tiny section of the literature, it is straightforward to disregard retractions as extreme outliers and focus instead of the 99.95% of unretracted articles in order to estimate the reliability of highly ranked journals.

## The Other 99.95% of the Literature

In the literature covering unretracted, peer-reviewed articles, one can identify at least eight lines of evidence suggesting that articles published in higher ranking journals are methodologically either not stronger or, indeed, weaker than those in lower ranking journals. In contrast, there is no evidence that articles published in higher ranking journals are methodologically stronger. Methodology here refers to several measures of experimental and statistical rigor with a potential bearing on subsequent replication or re-use. There is currently one article with evidence that higher ranking journals are better at detecting duplicated images (Bik et al., 2016).

In the following, I will quickly review the lines of evidence in the order of decreasing evidential strength.

### Crystallographic Quality

**Figure 1:**
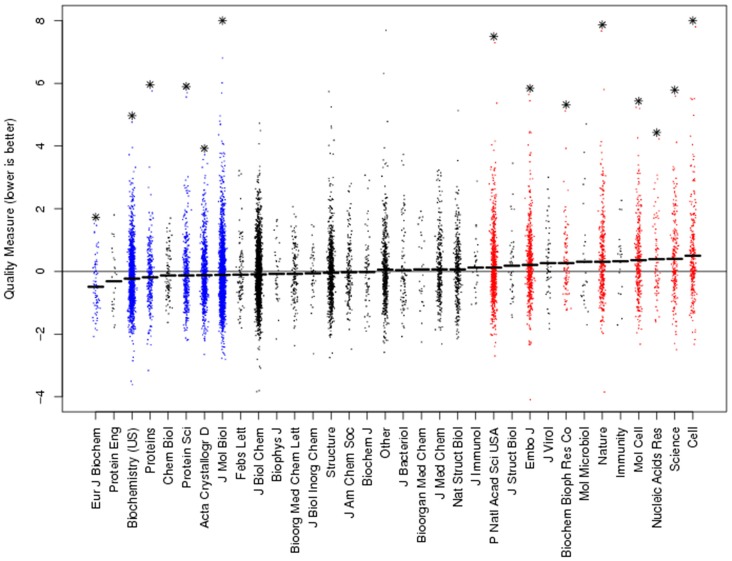
Ranking journals according to crystallographic quality reveals high-ranking journals with the lowest quality work. The quality metric (*y*-axis) is computed as a deviation from perfect. Hence, lower values denote higher quality work. Each dot denotes a single structure. The quality metric was normalized to the sample average and journals ranked according to their mean quality. Asterisks denote significant difference from sample average. Figure courtesy of Dr. Ramaswamy, methods in Brown and Ramaswamy (2007).

The quality of computer models of molecular structures, derived from crystallographic work, can be quantified by a method which includes the deviations from known atomic distances and other factors (Brown and Ramaswamy, 2007). Averaging the quality metric for each journal, high-ranking journals such as *Cell*, *Molecular Cell*, *Nature, EMBO Journal* and *Science* publish significantly substandard structures (Figure 1, courtesy of Dr. Ramaswamy, methods in Brown and Ramaswamy, 2007). The molecular complexity or the difficulty of the crystallographic work cannot explain this finding, as these factors are incorporated in the computation of the quality metric.

### Effect Sizes in Gene-Association Studies

**Figure 2:**
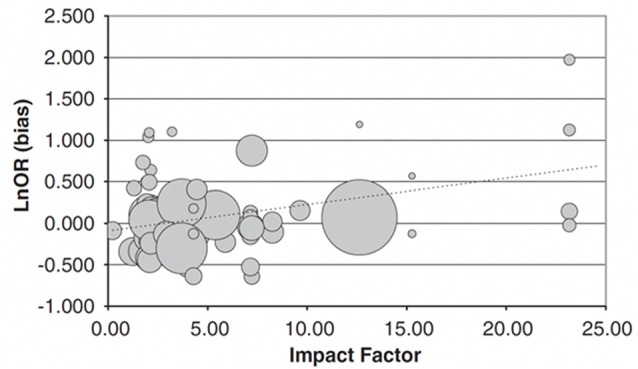
Relationship between impact factor (IF) and extent to which an individual study overestimates the likely true effect. Data represent 81 candidate gene studies of various candidate genes with psychiatric traits. The bias score (*y*-axis) represents the effect size of the individual study divided by the pooled effect size estimated indicated by meta-analysis, on a log-scale. Therefore, a value greater than zero indicates that the study provided an overestimate of the likely true effect size. This is plotted against the IF of the journal the study was published in (*x*-axis). The size of the circles is proportional to the sample size of the individual study. Bias score is significantly positively correlated with IF, sample size significantly negatively. Figure from Munafò et al. (2009).

Analyzing effect sizes in gene-association studies, Munafò et al. (2009) found that for 81 different studies on psychiatric traits, higher ranking journals overestimated the size of the gene-trait association, while the sample size decreased with increasing ranking of the journal (Figure 2). Phrased differently and more generally, inflated effect sizes are disproportionately often found in journals which rank more highly and publish studies with lower sample sizes.

### Statistical Power in Neuroscience/Psychology

**Figure 3:**
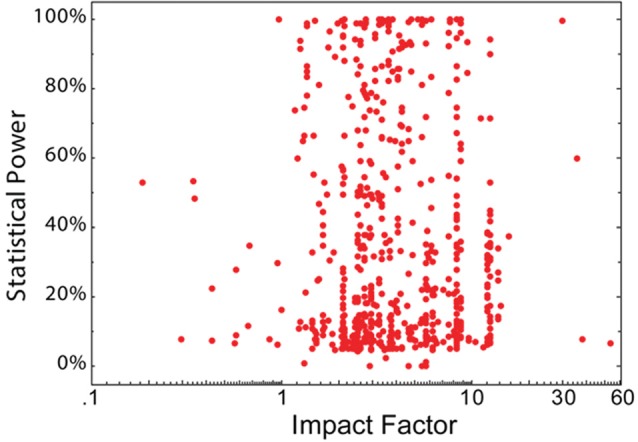
No association between statistical power and journal IF. The statistical power of 650 eligible neuroscience studies plotted as a function of the IF of the publishing journal. Each red dot denotes a single study. Figure from Brembs et al. (2013).

Statistical power (defined as 1—type II error rate; a measure computed from sample size and effect size) allows inference as to the likelihood that a nominally statistically significant finding actually reflects a true effect. As such, statistical power is directly related to the reliability of the experiments conducted. Button et al. (2013) analyzed the statistical power of 730 individual primary neuroscience studies. These data do not show any correlation with journal rank (Brembs et al., 2013; Figure 3).

Cognitive neuroscience and psychology also seem to suffer from insufficient statistical power. In their case, unlike in the neuroscience case, statistical power is even *negatively* correlated with journal rank (Szucs and Ioannidis, 2017). Taken together, these results suggest that in the covered fields at least, results from higher ranking journals tend to be less reliable than those from lower ranking journals, with a low overall reliability, as expressed in statistical power.

### Experimental Design in *in Vivo* Animal Experimentation

**Figure 4:**
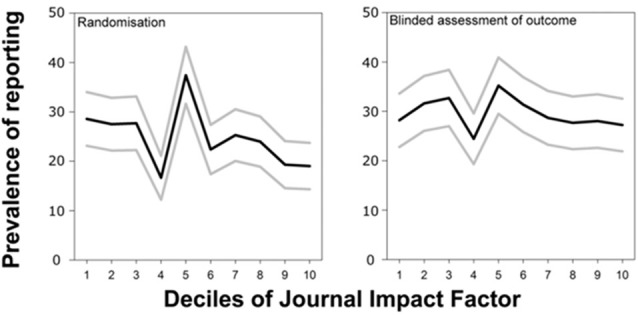
High ranking journals do not have a higher tendency to report more randomization nor blinding in animal experiments. Prevalence of reporting of randomization and blinded assessment of outcome in 2671 publications describing the efficacy of interventions in animal models of eight different diseases identified in the context of systematic reviews. Figure modified from Macleod et al. (2015).

In preclinical research studying animal models of disease, a widely used standard experimental design requires randomized assortment of animals into the treatment and control group, respectively, as well as an outcome assessment where the assessor scoring the outcome is blind as to the treatment group of the animal. Analyzing the methods sections of publications reporting *in vivo* experiments of animal disease models where this design should have been applied, Macleod et al. (2015) found that the prevalence of reporting randomization before the treatment correlated negatively with journal rank, while reporting of blind outcome assessment was not correlated (Figure 4, modified from Macleod et al., 2015). Inasmuch as this reporting correlates with actual experimentation, such publications in higher ranking journals would hence be less reliable than those in other journals: not reporting bias precludes replication and a reported bias may still entail that the bias created the observed effect. If randomization were not rarer (or even more frequent) in high vs. low-ranking journals, authors of articles in high-ranking journals are at least failing to report this randomization more often than authors in lower ranking journals.

### Errors in Genomics, Cognitive Neuroscience and Psychology

**Figure 5:**
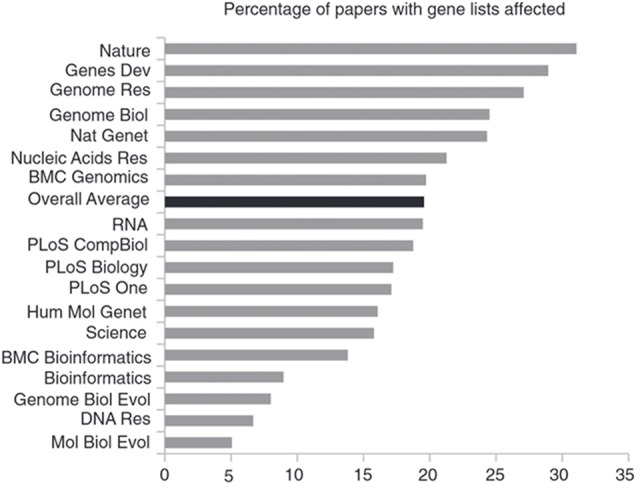
Journals with above-average error-rate rank higher than journals with a lower error-rate. Shown is the prevalence of gene name errors in supplementary Excel files as the percentage of publications with supplementary gene lists in Excel files affected by gene name errors. Figure modified from Ziemann et al. (2016).

With the advent of data deposition mandates by some journals, it has become a regular practice to supply the required data as supplemental files, often in spreadsheet form, compatible with the Excel software (Microsoft Corp., Redmond, WA, USA). However, when using Excel’s default settings, gene symbols and accession numbers may inadvertently be converted into dates or floating point numbers (Ziemann et al., 2016). These errors are widespread in the literature and the journals with above average error-rate are more highly ranked than journals with below-average error rate (Figure 5, modified from Ziemann et al., 2016). The authors speculate that the correlation they found is due to higher ranking journals publishing larger gene collections. This explanation, if correct, would suggest that, on average, error detection in such journals is at least not superior to that in other journals.

**Figure 6:**
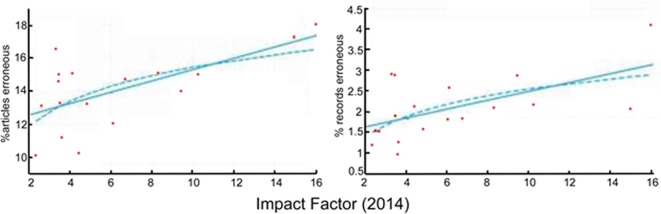
*p*-value reporting errors correlate significantly with journal rank. The correlation of the median percentage of articles with erroneous articles (left; which can contain multiple erroneous records) or individual records (right) in a given journal and journal IFs. Both linear and logarithmic (log[journal IF]) trend lines are shown. Figure redrawn from Szucs and Ioannidis (2016).

Another source of error in the literature are *p*-value reporting errors, i.e., the *p*-values reported in a publication deviate from the *p*-value calculated from the data. Comparing only those *p*-value reporting errors that changed the significance of the outcome across their sample of 18 journals in cognitive neuroscience and psychology, Szucs and Ioannidis (2016) found a significant correlation between the rate of such erroneous records and the IF of the journal (Figure 6, redrawn from Szucs and Ioannidis, 2016). Interestingly, the errors were highly skewed in the direction of reporting a non-significant computed *p*-value as significant (Szucs and Ioannidis, 2016).

### Criteria for Evidence-Based Medicine

One way of estimating the reliability of scientific publications is to count how many criteria for evidence-based medicine (e.g., according to JBJS-A, Australian National Health and Medical Research Council guidelines or GRADE) have been fulfilled and correlate these data with journal rank. There currently are four such studies, where two found such a correlation, indicating higher-ranked journals publish work where more criteria are fulfilled (Obremskey et al., 2005; Lau and Samman, 2007) and two did not (Bain and Myles, 2005; Tressoldi et al., 2013). In the case of these studies, if there is any actual correlation between journal rank and levels of evidence in medicine, it appears to be too weak to be detected consistently. For further discussion of these studies see Brembs et al. (2013).

### Reliability Metrics in Psychology

In the wake of recent replication efforts on psychology (Open Science Collaboration, 2015), metrics have been developed to detect tell-tale signs of questionable research practices or publication bias in a body of published work. These metrics commonly use *p*-value distributions or statistical power and other values which can be computed from the published articles to estimate the reliability of published research results. In a recent study (Bittner and Schönbrodt, 2017), the authors compared the *p*-values and statistical power in two psychology journals with very different IFs (0.79 vs. 5.03) for signs of p-hacking and other questionable research practices. In this study, a selection of three different metrics all indicate that the journal with the higher IF published less reliable results than the journal with the lower IF.

### Reproducibility Efforts

Current reproducibility efforts are comparatively small scale, with regard to the number of journals covered in each initiative, and hence cannot provide conclusive evidence as to any differences between journals in the reproducibility of the studies they publish. However, in studies where several journals were covered, the highest ranking journals did not stand out with a particularly high reproducibility, suggesting it may be only average in these journals (Scott et al., 2008; Prinz et al., 2011; Begley and Ellis, 2012).

## Conclusion

There are currently several lines of evidence in the literature, suggesting that highly prestigious journals fail to reach a particularly high level of reliability. On the contrary, some of the data seem to indicate that, on average, the highest ranking journals often struggle to raise above the average reliability levels set by the other journals (Table 1).

**Table 1:** Overview of the cited literature on journal rank and methodological soundness.

Field	Criteria	Outcome	References
Biomedicine	Image duplications	Higher ranking journals show a lower incidence of image duplications	Bik et al. (2016)
Crystallography	Quality of computer models	Five high-ranking journals significantly below average quality	Brown and Ramaswamy (2007)
Molecular psychiatry	Sample sizes and effect sizes	Higher ranking journals overestimated effect sizes with smaller sample sizes	Munafò et al. (2009)
Neuroscience, psychology	Statistical power	Either no correlation of journal rank with statistical power or a negative correlation	Brembs et al. (2013) Szucs and Ioannidis (2017)
*In vivo* animal experimentation in disease models	Reporting of randomization and blinded assessment of outcome	Lower reporting of randomization in higher ranking journals and no correlation with reporting of blinded assessment of outcome	Macleod et al. (2015)
Genomics, cognitive neuroscience and psychology	Gene name and *p*-value errors	More errors in higher ranking journals	Ziemann et al. (2016) Szucs and Ioannidis (2016)
Medicine	Criteria for evidence-based medicine	Two studies found that higher-ranking journals met more criteria, while two failed to detect such an effect	Obremskey et al. (2005) and Lau and Samman (2007) Bain and Myles (2005) and Tressoldi et al. (2013)
Psychology	Three reliability metrics: P-Curve, TIVA and R-index	All three metrics indicate that the higher ranking of two journals publishes less reliable work	Bittner and Schönbrodt (2017)
Biomedicine	Reproducibility of experiments	Reproducibility is low, not even “top” journals stand out	Scott et al. (2008), Prinz et al. (2011) and Begley and Ellis (2012)

*Applying criteria such as peer-review, methodology and spread of journal rank covered, the studies cited in the top six rows would be considered as better supported than the bottom three rows*.

In particular, comparing higher with lower ranked journals, two main conclusions can be drawn: (1) experiments reported in high-ranking journals are no more methodologically sound than those published in other journals; and (2) experiments reported in high-ranking journals are often less methodologically sound than those published in other journals.

Interestingly, not a single study provides evidence for the third option of higher-ranking journals publishing the **most** sound experiments. It is this third option that one would expect at least one area of inquiry to have conclusively demonstrated, if there was a true positive association between journal rank and reliability.

At the time of this writing, there is one single publication reporting that image duplication is lower in higher ranking journals (Bik et al., 2016). This result conflicts with the increased rate of fraud reported for higher ranking journals (Cokol et al., 2007; Fang and Casadevall, 2011; Fang et al., 2012). Potentially, these contradictory results may indicate that higher ranking journals may be more effective specifically in detecting duplicated images (perhaps due to a superior/more expensive software solution?), but failing in virtually all other aspects.

Importantly, these conclusions have been drawn from evidence collected from the published, unretracted literature, sampling many thousands of publications and data-sets from a variety of experimental fields. This method excludes differences in readership and associated scrutiny and directly approaches a reliability-based notion of methodological “quality”.

In total, none of the reported effects of journal rank, even those nominally significant, indicate a clear, obvious difference between journals. All journals seem to have difficulties coping with the issue of reliability, regardless of perceived prestige, regardless of the selectivity of the journal. Thus, the most conservative interpretation of the available data is that the reliability of scientific results does not depend on the venue where the results was published. In other words, the prestige, which allows high ranking journals to select from a large pool of submitted manuscripts, does not provide these journals with an advantage in terms of reliability. If anything, it may sometimes become a liability for them, as in the studies where a negative correlation was found. This insight entails that even under the most conservative interpretation of the data, the most prestigious journals, i.e., those who command the largest audience and attention, at best excel at presenting results that *appear* groundbreaking on the surface. Which of those results will end up actually becoming groundbreaking or transformative, rather than flukes or frauds, is a question largely orthogonal to the journal hierarchy.

It is up to the scientific community to decide if the signal-to-noise ratio in these journals is high enough to justify the cost of serial scandals and, in the case of medical journals, loss of life, due to unreliable research.

This body of evidence complements evolutionary models suggesting that using productivity as selection pressure in hiring, promotion and funding decisions leads to an increased frequency of questionable research practices and false positive results (Higginson and Munafò, 2016; Smaldino and McElreath, 2016). Arguably, scientists who become “successful” scientists by increasing their productivity through reduced sample sizes (i.e., as a consequence, reduced statistical power) and by publishing in journals with a track record of unreliable science, will go on teaching their students how to become successful scientists. Already after only one generation of such selection pressures, we begin to see the effects on the reliability of the scientific literature. If scholars strive to convince the public that the scientific endeavor deserves its trust and funds, abolishing these selection pressures is likely the most urgent evidence-based policy of any current reform efforts.

## Author Contributions

The author confirms being the sole contributor of this work and approved it for publication.

## Conflict of Interest Statement

The author declares that the research was conducted in the absence of any commercial or financial relationships that could be construed as a potential conflict of interest.
