# Corrigendum: Prestigious Science Journals Struggle to Reach Even Average Reliability

**DOI:** 10.3389/fnhum.2018.00376

**Published:** 2018-10-09

**Authors:** Björn Brembs

**Affiliations:** Institute of Zoology—Neurogenetics, Universität Regensburg, Regensburg, Germany

**Keywords:** journal ranking, journals, reliability, reproducibility of results, science policy

In the original article, there were several errors.

A correction has been made to *Introduction*, paragraph one:

The most groundbreaking, transformative research results deserve a broad readership and a large audience. Therefore, scientists submit their best work to the journals with the largest audience. While the number of scientists has been growing exponentially over the last decades, the number of journals with a large audience has not kept up, neither has the number of articles published per journal. Consequently, the acceptance rates for the most prestigious journals have fallen below 10% and the labor of rejecting submissions has become these journals' largest cost item. Assuming that this exclusivity allows the journals to separate the wheat from the chaff, successful publication in these journals is treated as a quality signal in hiring, promotion, and funding decisions. If anything, these developments have fueled the circularity of this relationship: today, publishing ground-breaking science in a high ranking journals is not only important for science to advance but also for an author's career to advance. Even before science became hypercompetitive at every level, now and again results published in prestigious journals were later found to be false. This is the nature of science. Science is difficult, complicated, and perpetually preliminary. Science is self-correcting and better experimentation will continue to advance science to the detriment of previous experiments. Today, however, fierce competition exacerbates this trait and renders it a massive problem for scholarly journals. Now it has become their task to find the ground-breaking among the too-good-to-be-true data, submitted by desperate scientists, who face unemployment and/or laboratory closure without the next high-profile publication. This is a monumental task, given that sometimes it takes decades to find that one or the other result rests on flimsy grounds. How is our hierarchy of more than 30,000 journals holding up?

A correction has been made to *Introduction*, paragraph two:

At first glance, it appears as if our journals fail miserably. Evaluating retractions, the capital punishment for articles found to be irreproducible, it was found that the most prestigious journals boast the largest number (Fang and Casadevall, 2011) and that most of these retractions are due to fraud (Fang et al., 2012). However, data on retractions suffer from two major flaws which make them rather useless for answering questions about the contribution of journals to the reliability (or lack thereof) of our scholarly literature: (1) retractions cover only about 0.05% of the literature; and (2) they are confounded by error-detection variables that are hard to trace. So maybe our journals are not doing so horribly after all?

A correction has been made to *Statistical Power in Neuroscience/Psychology*, paragraph one:

Statistical power (defined as 1—a, where a denotes the type II error rate; a measure computed from sample size and effect size) allows inference as to the likelihood that a nominally statistically significant finding actually reflects a true effect. As such, statistical power is directly related to the reliability of the experiments conducted. Button et al. (2013) analyzed the statistical power of 730 individual primary neuroscience studies. These data do not show any correlation with journal rank (Brembs et al., 2013; Figure 3).

The author apologizes for these errors and states this corrigendum does not change the scientific conclusions of the article in any way.

The original article has been updated.

## Conflict of interest statement

The author declares that the research was conducted in the absence of any commercial or financial relationships that could be construed as a potential conflict of interest.
